# Adsorption of tetrakis(4-sulfophenyl)porphyrin onto liposomal surfaces composed of neutral diacylphosphatidylcholine and release by cyclodextrin†

**DOI:** 10.1039/c8ra01411f

**Published:** 2018-03-27

**Authors:** Yuki Tsuchiya, Toshimi Nakaya, Tomoyuki Kakigi, Kouta Sugikawa, Atsushi Ikeda

**Affiliations:** Department of Applied Chemistry, Graduate School of Engineering, Hiroshima University 1-4-1 Kagamiyama Higashi-Hiroshima 739-8527 Japan

## Abstract

Anionic tetrakis(4-sulfophenyl)porphyrin (TPPS) interacts with liposomal surfaces composed of neutral diacylphosphatidylcholine at high lipid concentrations. TPPS interacted with liposomal surfaces through four contact points. The association constant was obtained to be 9.0 × 10^5^ M^−4^. TPPS was peeled off the liposomal surfaces by the addition of cyclodextrin.

## Abstract 2

Interactions between tetrakis(4-sulfophenyl)porphyrin and liposomal surfaces with neutral diacylphosphatidylcholine occurred and can be controlled by the addition of cyclodextrin.

## Graphical abstract

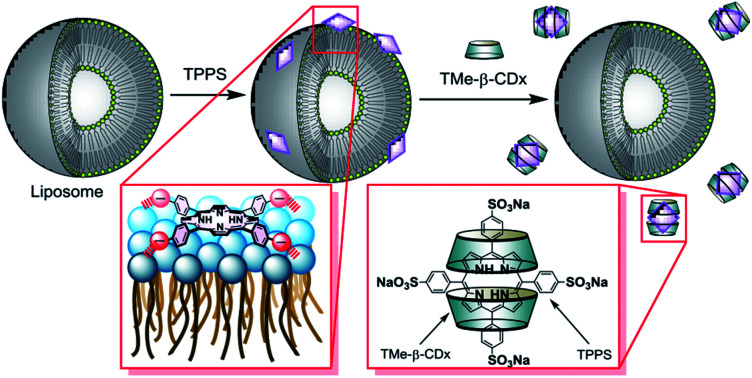

## Introduction

**Fig. 1:**
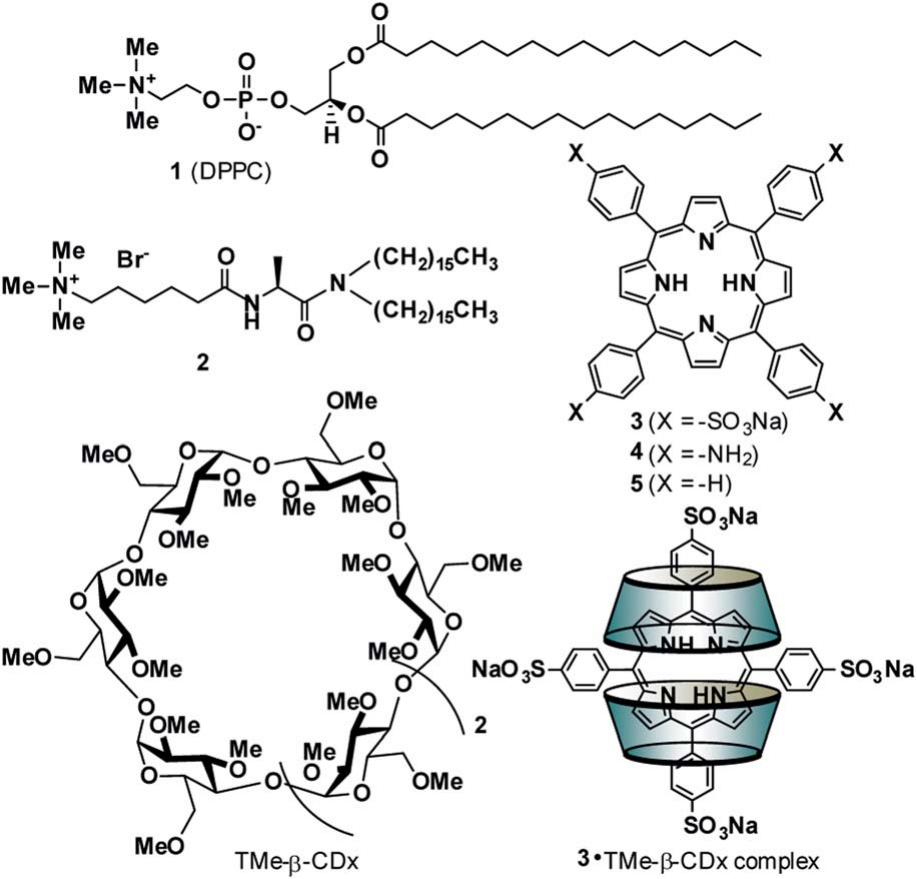
Compound structures and schematic illustrations of the 3·TMe-β-CDx complex.

**Scheme 1:**
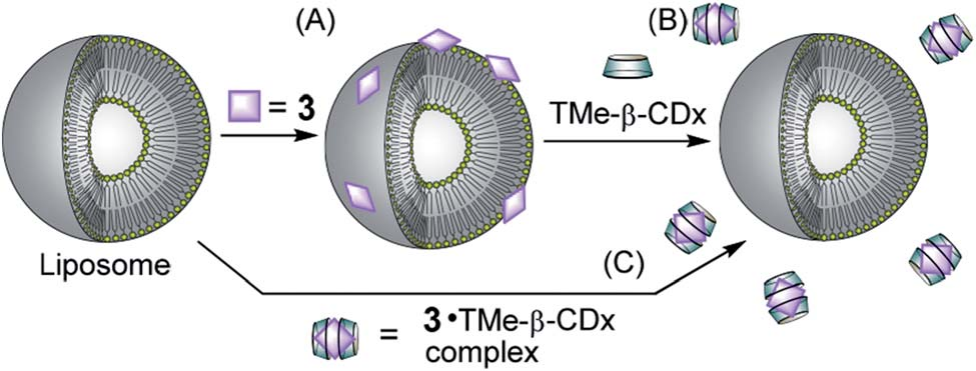
Schematic illustrations of (A) adsorption of 3 on the liposomal surface, (B) exfoliation of 3 by TMe-β-CDx and (C) no interaction between the 3·TMe-β-CDx complex and a liposome.

The adsorption of compounds such as polymers and nanoparticles onto the cell surface is important in developing drug carriers^1–5^ and functional materials.^6–12^ Recently, several groups reported that polyelectrolytic biomacromolecules, such as DNA or nanoparticles with anionic surfaces, were able to adsorb onto liposomal surfaces composed of neutral diacylphosphatidylcholine (PC).^1–12^ Although the details of the interactions remain unclear, multipoint interactions might exist between the negative charges of these compounds or materials and the positive charge N^+^ of the P^−^–N^+^ (phosphorous–nitrogen) dipole of PC.^13,14^ Recently, we showed that 5,10,15,20-tetrakis(4-sulfophenyl)porphyrin (3) (Fig. 1) interacts with the liposomal surface by formation of one-dimensional self-assembled structures (J-aggregates) under acidic conditions.^15,16^ In contrast, neutralization of the solution deformed the porphyrin J-aggregates, leading to release of 3 from the liposomal surface.^15,16^ These interactions are important not only for development of novel functional materials by using liposomes, but also for internalization into cells by endocytosis as the first step of intracellular uptake. In this report, 3 was found to interact with liposomal surfaces composed of neutral lipids at high liposome concentrations without the formation of porphyrin J-aggregates (Scheme 1A). Furthermore, 3 was released from the liposomal surfaces by addition of trimethyl-β-cyclodextrin (TMe-β-CDx) (Fig. 1, Scheme 1B).

## Results and discussion

### Interaction between anionic porphyrin and neutral lipid

**Fig. 2:**
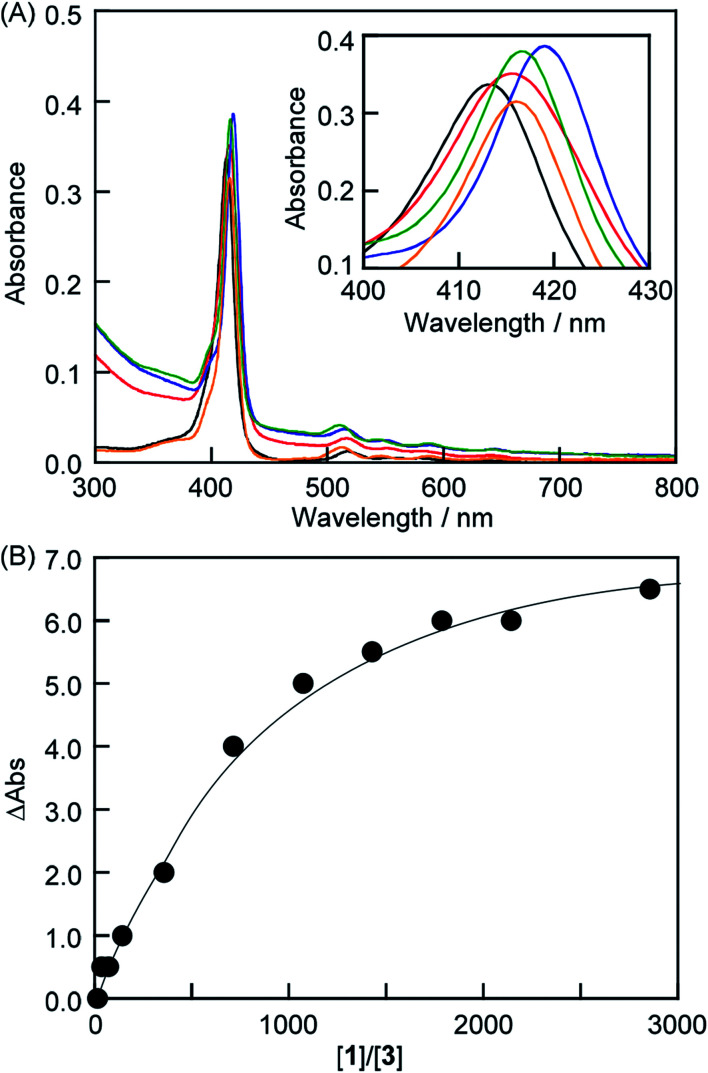
(A) UV-vis absorption spectra of 3 (black), a mixture of 3 and liposome-1 (red), a mixture of 3 and liposome-1–2 (blue), a mixture of 3, liposome-1 and TMe-β-CDx (green), and the 3·TMe-β-CDx complex (orange) {1 mm cell, [3] = 7.0 μM, [1] = 10.0 mM in phosphate buffer (pH = 6.8)}. (B) *λ*_max_ shift of 3*versus* concentrations of 1 at 20 °C {[3] = 7.0 μM, [1] = 0.5–20.0 mM in phosphate buffer (pH = 6.8)}.

The interactions between anionic porphyrin 3 and the neutral lipid 1 [1,2-dipalmitoyl-*sn-glycero*-3-phosphocholine (DPPC), Fig. 1] were investigated. Concentration dependent UV-vis absorption spectra of 3 were measured by the addition of liposomes composed of 1 (liposomes-1) (Fig. 2) and the red shift in the Soret band was observed at 20 °C (413 → 415.5 nm, ΔAbs = 2.5 nm). The red shift was not because of the formation of one-dimensional self-assembled structures because: (i) 3 cannot form self-aggregates under neutral conditions because protonation of 3 is essential for self-association, and (ii) the shift value is too small for self-aggregates because the Soret band of the porphyrin J-aggregates displayed a significant red shift to 491 nm.^15–18^ If sulfo groups of 3 interact with ammonio groups of 1 on the liposomal surface, the addition of cationic lipids in the liposome should facilitate the formation of strong interactions between anionic 3 and the liposomal surface *via* electrostatic interactions.^19–21^ Therefore, a cationic lipid (2) was mixed with lipid 1 in the liposomes {[1] : [2] = 7 : 3 (mol mol^−1^)}. As shown in Fig. 2A (blue line), the *λ*_max_ of 3 (419 nm) was red-shifted by 6 nm when compared with the results obtained by 3 alone in the absence of liposomes [Fig. 2A (black line)]. Therefore, the red shift suggests an interaction between 3 and lipid 1. The shifts of Δ*λ*_max_ in the Soret band of 3 were plotted against the concentration of 1 in Fig. 2B. We were not able to determine the association constant between 3 and lipid 1 from a curve in Fig. 2B. The association constant determined from ^1^H NMR spectrum is described below. In contrast, we investigated interactions between cationic porphyrin 4 (Fig. 1) and lipid 1. Although the absorbance of 4 increased because of the light scattering of liposomes-1, no shift of Δ*λ*_max_ in the Soret band of 4 was observed (Fig. S1†), indicating that 4 did not interact with the liposomal surface.

### Existence of porphyrin on the liposomal surface

**Fig. 3:**
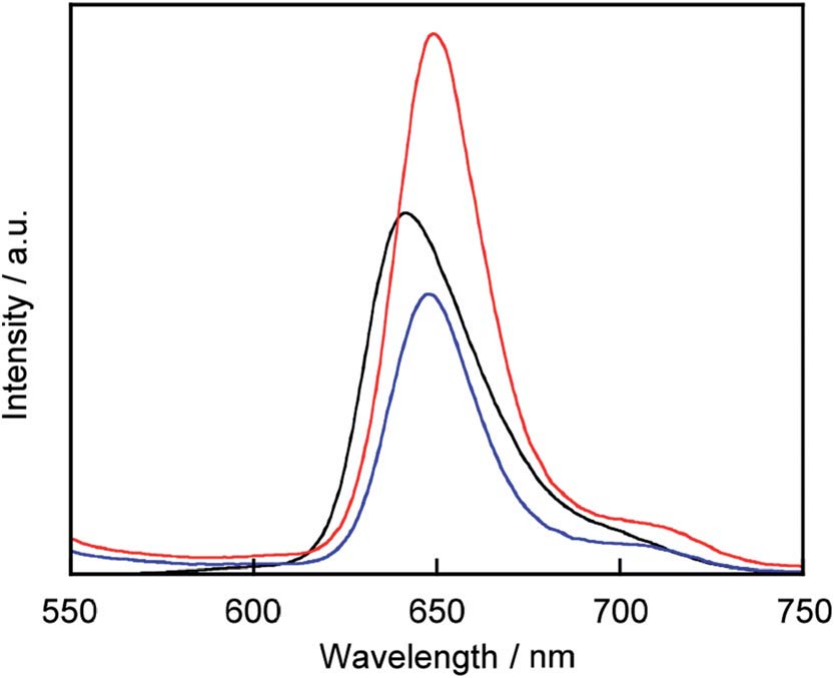
Fluorescence spectra of 3 (black), a mixture of 3 and liposome-1 (red), and a mixture of 3 and LMIC_60_ (blue). Excitation wavelength: 517 nm. [3] = 0.05 mM, [1] = 5.0 mM and [C_60_] = 0.25 mM in phosphate buffer (pH = 6.8).

C_60_ is known to act as a quencher in liposomes.^22^ To confirm that 3 exists on the liposomal surface, we measured fluorescence quenching of 3 by C_60_ in lipid-membrane-incorporated C_60_ (LMIC_60_, Fig. 3). The presence of C_60_ led to a fluorescence quenching of 48%, indicating that 3 exists in the neighborhood of C_60_.

### Form of porphyrin on the liposomal surface

**Fig. 4:**
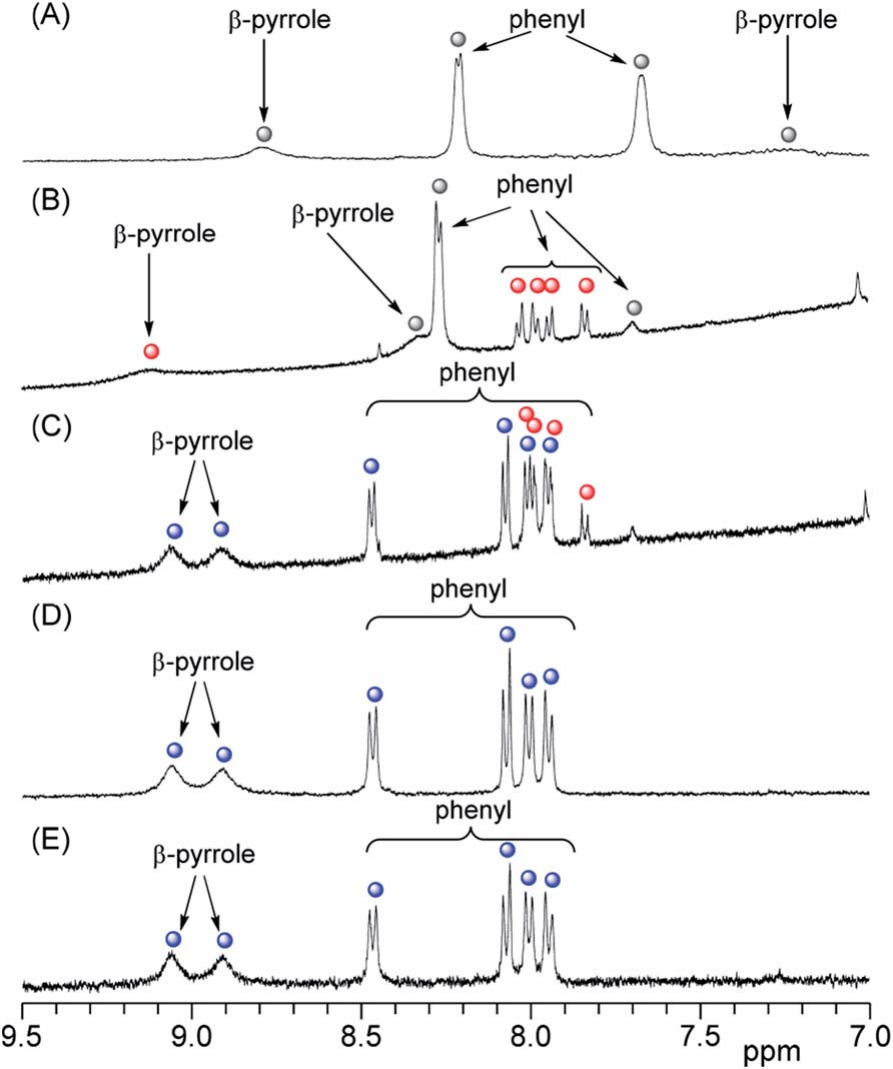
Partial ^1^H NMR spectra of (A) 3 ([3] = 0.4 mM), (B) the mixture of 3 and liposome-1 ([3] = 0.05 mM and [1] = 25 mM), (C) the mixture of 3, liposome-1 and TMe-β-CDx ([3] = 0.05 mM, [1] = 25 mM and [TMe-β-CDx] = 1.0 mM), (D) the 3·TMe-β-CDx complex ([3·TMe-β-CDx complex] = 0.40 mM) and (E) the mixture of the 3·TMe-β-CDx complex and liposome-1 ([3·TMe-β-CDx complex] = 0.20 mM and [1] = 4.0 mM) in D_2_O-phosphate buffer (pH = 6.8) (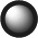: free 3; 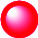: 3 on the liposomal surface; 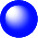: 3 in the 3·TMe-β-CDx complex).

**Scheme 2:**
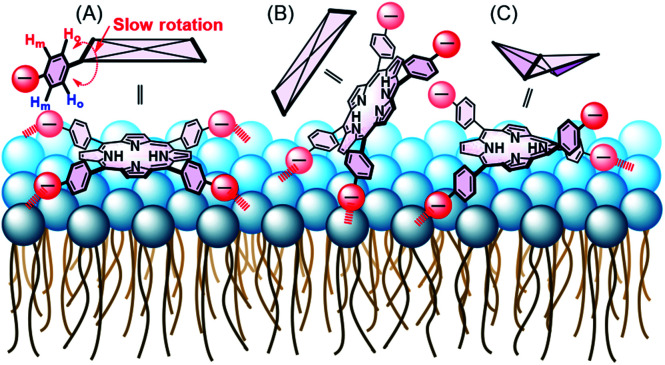
Schematic illustration of (A) four point interactions between 1 and 3, (B) two point interactions between 1 and the *cis*-position of 3 and (C) two point interactions between 1 and the *trans*-position of 3 with a saddle-shape structure. Red arrows show the distortion of the lipid membranes and red broken lines show the interactions.

In the ^1^H NMR spectra, 3 gives rise to two peaks arising from the phenyl protons in the *ortho* and *meta* positions because of the *D*_4h_ symmetry of 3 (Fig. 4A and S2A†). Although the peak assigned to the phenyl protons in the *meta* position (8.2 ppm) appeared as a sharp doublet, the corresponding peak for the protons in the *ortho* position (7.7 ppm) was broader and this line-broadening was ascribed to self-aggregation of 3.^23,24^ In Fig. 4B, the phenyl protons in the *ortho* positions of 3 were observed as a pronounced broaden peak (grey circle).^23^ In contrast, when 3 interacted with liposome-1, two pairs of phenyl protons in the *ortho* and *meta* positions appeared in a 1 : 1 ratio [Fig. 4B (red circles) and S2B†]. The result suggests the following three models for the interaction between 1 and 3: *C*_4v_, *C*_2v_ and *C*_2v_ symmetries, depending on the interaction on the liposomal surface (Scheme 2). In Scheme 2A, 3 interacts with liposomal surfaces by four point interactions. The porphyrin face of 3 is shaped asymmetrical at the upper and lower sides. If the rotation of phenyl units decreases and is slower than the NMR time scale because of steric hindrance by the liposome surface, the two *ortho* and two *meta* protons in one phenyl unit are in different chemical environments (see Scheme 2A) and give rise to the four signals in Fig. 4B (red circles). For the other possible models, 3 interacts with the liposomal surface by only two SO_3_^−^ in the *cis*-position with *C*_2v_ (Scheme 2B) or the *trans*-position with *C*_2v_ (Scheme 2C), in which 3 interacts with the N^+^ of P^−^–N^+^ in liposome-1. Because 3 has different symmetries in the three models, the β-pyrrole protons should appear as one, four or two sets of peaks in the NMR spectrum for models in Schemes 2A, B and C, respectively. As shown in Fig. 4B (red circle), the β-pyrrole protons appeared as a single broad peak, suggesting that the model in Scheme 2A is correct.

### Association constant between anionic porphyrin and neutral lipid

As shown in Scheme 2A, porphyrin 3 interacted with the liposome surface consisted of lipids 1 through four point interactions. Therefore, the equilibrium is defined as (1):

1 3 + 4·1 ⇌ 3 − 4·1

The concentrations of free 3 and 3 on the liposomal surface were determined by the peak intensities in Fig. 4B. When these values were substituted into eqn (2), we obtained the association constant (*K*_a_) = 9.0 × 10^5^ M^−4^.2
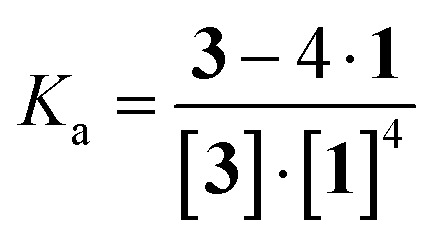


Porphyrin derivatives can form a 1 : 2 complex with TMe-β-CDx.^24–26^ Furthermore, all of the tetraphenylporphyrin (5) were released from the TMe-β-CDx cavities and transferred to the lipid membrane after mixing the 5·TMe-β-CDx complex with liposome-1 at 30 °C for 1 h.^25^ Therefore, we attempted the exchange reaction of 3 from TMe-β-CDx cavities to lipid membranes or the formation of direct interactions between liposome surfaces and the 3·TMe-β-CDx complex. After mixing the 3·TMe-β-CDx complex with liposome-1 under the same conditions reported previously,^25^ all peaks assignable to TMe-β-CDx in the 3·TMe-β-CDx complex remained in the ^1^H NMR spectrum (blue circles in Fig. 4D, E, S2D and E†). The result shows that porphyrin 3 did not transfer from the TMe-β-CDx cavities to the liposomes and the porphyrin remained in the TMe-β-CDx cavities (Scheme 1C). This observation suggests that 3 is too hydrophilic to incorporate into the hydrophobic lipid membrane. Furthermore, no chemical shift changes of these peaks indicate that the 3·TMe-β-CDx complex did not interact with the liposome surface by only a single point interaction because of steric hindrance by the two TMe-β-CDxs. Because *K*_a_ for the four point interactions was 9.0 × 10^5^ M^−4^, *K*_a_' per a single point interaction was estimated to be approximately 30 M^−1^ at most even if entropy and enthalpy gains by multi point interactions were excluded. Therefore, the 3·TMe-β-CDx complex cannot interact with the liposome surface by only a single point interaction. Consequently, the interactions between 3 and 1 need at least four point interactions.

### Exfoliation of porphyrin from the liposome surface by cyclodextrin

To control the association–dissociation of 3 onto the liposomal surface, TMe-β-CDx was added to the mixture of 3 and liposome-1. After adding TMe-β-CDx, although the *λ*_max_ of the Soret band of 3 is barely shifted (*i.e.*, 415 to 416 nm), the *λ*_max_ in the Q-band of 3 is shifted from 517 to 511 nm. These *λ*_max_ were the same as that observed for the 3·TMe-β-CDx complex (416 and 511 nm) (Fig. 2A, green and orange). Furthermore, peaks were observed in the ^1^H NMR spectrum for the 3·TMe-β-CDx complex (Fig. 4C and S2C†). The results show that most of 3 peeled off the liposomal surface and had formed a complex with two TMe-β-CDxs (Scheme 1B).

## Conclusions

In summary, anionic 3 was adsorbed onto liposomal surfaces composed of neutral lipid 1 at high lipid concentrations. The interaction of 3 with 1 was *via* four contact points. In contrast, the 3·TMe-β-CDx complex cannot be adsorbed onto liposomal surfaces because 3 interacts with 1 by only a single point and is encapsulated by two TMe-β-CDxs. Addition of TMe-β-CDx caused the release of 3 from the liposomal surface and complex formation with two TMe-β-CDxs. Consequently, the association–dissociation of 3 onto the liposomal surfaces can be controlled by the addition of TMe-β-CDx.

## Experimental

### Experimental materials

Trimethyl-β-cyclodextrin (TMe-β-CDx) and 5,10,15,20-tetrakis(4-sulfophenyl)porphyrin (3) were purchased from Wako Pure Chemical Industries Ltd (Tokyo, Japan) and Tokyo Chemical Industries Co., Ltd (Tokyo, Japan), respectively. 1,2-Dimyristoyl-*sn-glycero*-3-phosphocholine (DMPC, 1) was obtained from Funakoshi Co., Ltd (Tokyo, Japan). Compound 2 was prepared according to the method described previously.^27^

### Phosphate buffer

A phosphate buffer was prepared by dissolving a mixture of NaH_2_PO_4_·2H_2_O (390 mg, 2.50 mmol), Na_2_HPO_4_·12H_2_O (181 mg, 0.50 mmol) and K_2_SO_4_ (1.70 mg, 9.74 mmol) in pure water or D_2_O (100 mL), to reach a final pH of 6.5 at 23 °C.

### Preparation of liposome-1 and liposome-1–2

An appropriate amount of 1 or a mixture of 1 and 2 ([1] : [2] = 7 : 3 mol mol^−1^) was dissolved in chloroform. The solvent was evaporated under a gentle stream of nitrogen, followed by a period under vacuum to remove any traces of solvent. The resulting thin lipid films were hydrated on the wall of the vial above the phase transition temperature with an appropriate amount of phosphate buffer. The hydrated materials were subjected to eight freeze–thaw cycles (−195 and 50 °C) to give unilamellar vesicles, which were extruded 11 times through 0.05 μm pores using a LiposoFast miniextruder from Avestin (Ottawa, Canada) above the phase transition temperature. The resulting liposomes were uniform in size with a diameter of approximately 80 nm. The final lipid concentration was 3.0 mM.

### Preparation of the 3·TMe-β-CDx complex

Compound 3 (5.00 mg, 5.26 × 10^−6^ mol) and TMe-β-CDx (15.0 mg, 1.05 × 10^−5^ mol) were placed in an agate capsule with two agate-mixing balls. The resulting mixture was agitated vigorously at 30 Hz for 20 min using a high-speed vibration mill (MM 200; Retsch Co., Ltd., Haan, Germany). The solid mixture was suspended in either phosphate buffer or D_2_O–phosphate buffer (1.5 mL) to produce a dark purple emulsion. Subsequent centrifugation (18 000 × *g*, 25 °C, 20 min) removed non-dispersed 3 from the solution. The concentration of 3 in the 3·TMe-β-CDx complex was determined to be 0.03 mM by measuring the absorbance of the solution at *λ*_max_ in water. The molar absorption coefficient for the water-soluble 3·TMe-β-CDx complex is *ε*_416_ = 3.79 × 10^5^ dm^3^ mol^−1^ cm^−1^.

### Spectrophotometric assay

The absorbance spectra were scanned using a UV-2550 spectrophotometer (Shimadzu Corporation, Kyoto, Japan). Fluorescence spectra were obtained using an LS 55 luminescence spectrometer (Perkin-Elmer, Waltham, MA, USA). The excitation and emission wavelengths were 517 and 600–750 nm, respectively.

## Conflicts of interest

There are no conflicts to declare.

## Supplementary Material

RA-008-C8RA01411F-s001
