# Supplementary material for: Adsorption of tetrakis(4-sulfophenyl)porphyrin onto liposomal surfaces composed of neutral diacylphosphatidylcholine and release by cyclodextrin
Source: RSC Adv. 2018 Mar 27;8(22):11930–4. doi: 10.1039/c8ra01411f (PMC9079261; doi:10.1039/c8ra01411f)
Supplement: RA-008-C8RA01411F-s001 [file RA-008-C8RA01411F-s001.pdf]

Electronic Supplementary Material (ESI) for RSC Advances.
This journal is © The Royal Society of Chemistry 2018

Electronic Supplementary Information

Adsorption of tetrakis(4-sulfophenyl)porphyrin onto liposomal surfaces composed of neutral diacylphosphatidylcholine and release by cyclodextrin

Yuki Tsuchiya, Toshimi Nakaya, Tomoyuki Kakigi, Kouta Sugikawa and Atsushi Ikeda

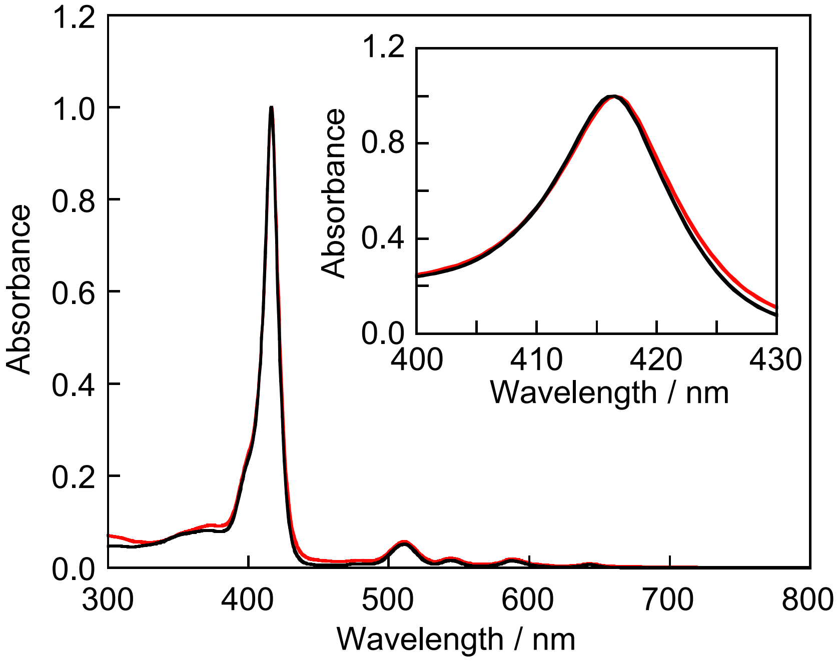

Fig. S1 UV-vis absorption spectra of the **4**•TMe-β-CDx complex in the absence (black) and in the presence (red) of liposome-**1** at 25 °C. The inset shows the region of 400–430 nm.

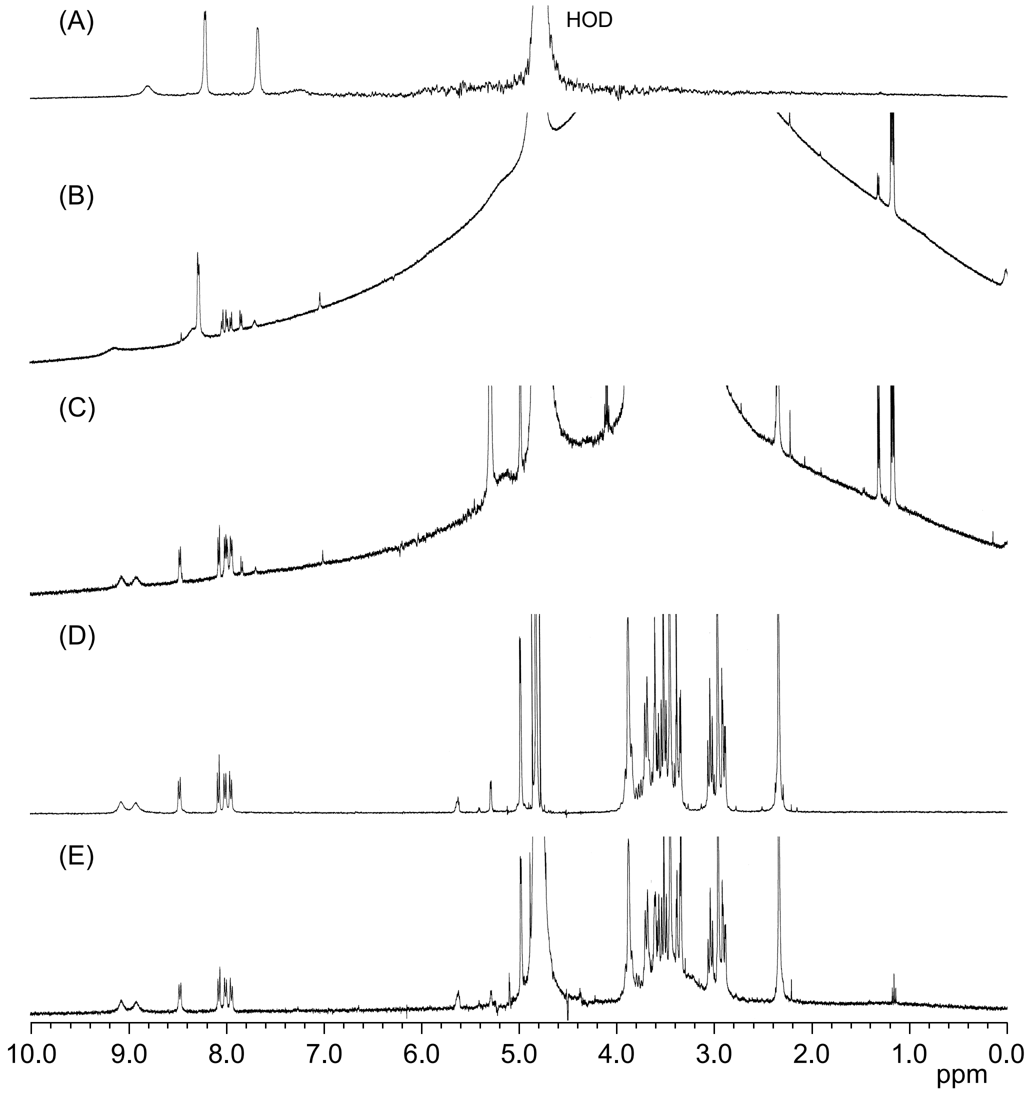

Fig. S2 Complete ^{1}H NMR spectra of (A) **3** ([**3**] = 0.4 mM), (B) the mixture of **3** and liposome-**1** ([**3**] = 0.05 mM and [**1**] = 25 mM), (C) the mixture of **3**, liposome-**1** and TMe-β-CDx ([**3**] = 0.05 mM, [**1**] = 25 mM and [TMe-β-CDx] = 1.0 mM), (D) the **3**•TMe-β-CDx complex ([**3**•TMe-β-CDx complex] = 0.40 mM) and (E) the mixture of the **3**•TMe-β-CDx complex and liposome-**1** ([**3**•TMe-β-CDx complex] = 0.20 mM and [**1**] = 4.0 mM) in the D_2O-phosphate buffer (pH = 6.8).
